# Effects of Laser Irradiation in High-Speed Gas Flow for Surface Treatments of Copper

**DOI:** 10.3390/mi15111296

**Published:** 2024-10-25

**Authors:** Mohamed Ezzat, Constantin Aniculaesei, Joong Wook Lee, Seong Ku Lee

**Affiliations:** 1 Center for Relativistic Laser Science, Institute for Basic Science (IBS), Gwangju 61005, Republic of Korea; mezzat@lira.bsu.edu.eg (M.E.); laserisfun@gmail.com (C.A.); 2 Department of Physics and Optoelectronics Convergence Research Center, Chonnam National University, Gwangju 61186, Republic of Korea; 3 Laser Institute for Research and Application (LIRA), Beni-Suef University, Beni-Suef 62511, Egypt; 4 Advanced Photonics Research Institute, Gwangju Institute of Science and Technology (GIST), Gwangju 61005, Republic of Korea

**Keywords:** laser treatment, Cu, supersonic nozzle, gas flow, microhardness

## Abstract

In this study, the impacts of laser irradiation on the surface morphology and hardness of copper (Cu) are investigated under various environments, including air, vacuum, and high-pressure gas flow through a supersonic nozzle. After irradiating Cu targets with laser pulses with energy of 30, 60, and 90 mJ/pulse, the surface structures of the targets are analyzed by scanning electron microscopy (SEM) and X-ray diffraction (XRD). The SEM analysis reveals diverse surface morphologies, including micro-cones, cavities, droplets, ripples, and island-like structures, depending on laser energy and environments. The XRD analysis provides insights into the structural changes induced by laser irradiation. The results indicate a significant enhancement in microhardness by a factor of 2.77, which is attributed to the surface and structural modifications incurred under various environments. In addition, the XRD analysis reveals a shift in the residual stress in the surface layers of copper from tensile before laser irradiation to compressive afterwards, highlighting the effectiveness of laser surface treatment in inducing favorable mechanical properties.

## 1. Introduction

The surface properties of materials have a significant impact on their performance and durability. Failures caused by wear, corrosion, and fatigue often originate from the material surface. The enhancement of surface integrity has, thus, become increasingly important in many applications. By improving the surface properties, materials can be made to withstand harsh environments more effectively and experience less wear and tear. While various techniques can be utilized to enhance the surface properties and optimize the overall material performance [1,2,3,4,5,6], the laser has great advantages as a tool for material processing due to its ability to modify the surface composition and microstructure and to develop unique phases [7,8], by appropriately controlling laser processing parameters [9,10,11]. Indeed, the unique capabilities of high-power lasers have led to rapid advancements in laser–matter interactions for material processing in recent decades [12,13]. As a result, applications of laser treatment have expanded from low-volume parts to higher-volume components such as hip implants [14], rotor components [15], turbine blades and discs [16], gear shafts [17], and bearing components [18]. Consequently, there is an increasing need to understand laser–material interaction phenomena in order to achieve better control and quality of desired features.

The use of laser radiation to modify the surface structure of a material is a complex process that involves a chain of intricate mechanisms, including the ablation mechanism and its threshold, the transport of laser energy, and the hydrodynamics and evolution of the plume. Furthermore, the ambient conditions and the surface roughness of the material have significant impacts on the interactions and energy deposition mechanisms during the laser-treatment process [19,20,21,22,23]. The control of laser surface treatment conditions can play important roles in optimizing the material performance [24].

Due to its remarkable electrical and thermal conductivities, excellent ductility, and resistance to atmospheric corrosion, copper (Cu) is a versatile metal with extensive industrial applications, including bearings, brushes, electrical sliding contacts, and resistance-welding electrodes. Nevertheless, its hardness and wear resistance are not high enough to meet certain performance requirements. To address this limitation, laser surface alloying is commonly used [25], with alloying components, such as tungsten (W) [26,27], chromium (Cr) [28,29], titanium (Ti) [30,31] nickel–titanium (NiTi) [32], as well as composites such as chromium/titanium/carbon nanotube (Cr/Ti/CNT) [33]. Recently, Shazia et al. reported the microhardness enhancement of copper irradiated by a 1064 nm laser under Ar and Ne environments, respectively [34]. In the present study, the feasibility of surface structuring via laser irradiation of copper under a high-pressure gas flow through a supersonic nozzle is explored, and the effects of laser irradiation on the material hardness are investigated under various laser energies and environments, including air, vacuum, and high-pressure gas flow through a supersonic nozzle with a backing pressure of 0.5, 1.0, and 1.5 bar. After irradiation, a significant increase in microhardness by a factor of 2.77 is observed under high-pressure gas flow through a supersonic nozzle.

## 2. Experimental

**Figure 1:**
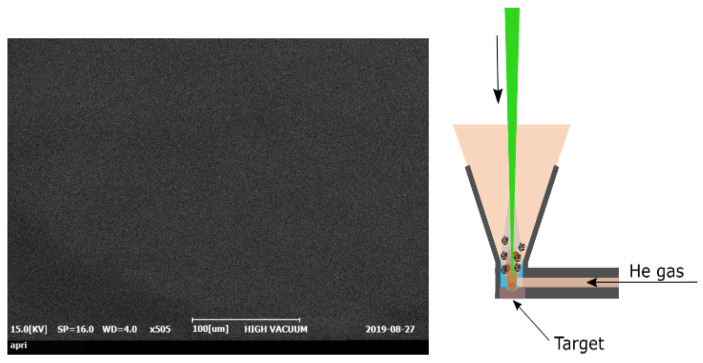
SEM images of the pristine surface and the schematic of the laser surface irradiation experiment. The direction of the laser radiation is perpendicular to the direction of the high-speed gas flow.

The experiment was conducted using a copper target (CU-113551), obtained from Nilaco, Japan, with a surface area of 1.5 × 1.5 cm^2^ and a thickness of 2 mm. The copper target was subjected to ultrasonic cleaning for ~30 min before the experiment. The chemical composition of the pristine Cu was analyzed via scanning electron microscopy with energy-dispersive X-ray spectroscopy (SEM-EDS, SEM, CX-100S, Coxem Co., Ltd., Republic of Korea), and the results are listed in Table 1. Figure 1 presents SEM images of the unprocessed Cu alongside the schematic diagram of the experiment.

The laser treatment was conducted using the high-vacuum chamber shown schematically in a previous paper [35]. To obtain accurate and reliable results, variables such as laser power and gas pressure were meticulously controlled. A dry pump and a turbo-pump were used to achieve a high vacuum of $5\times 10^{-4}\;torr$. Helium (He) gas was introduced into the side of a de Laval-type conical supersonic nozzle through a 2 mm diameter pipe at various backing pressures of 0.5, 1.0, and 1.5 bar. The flow of He was regulated via a pulsed solenoid valve (Parker Series 9) with an opening time of 6 ms. A time delay of +3 ms existed between the laser and the gas operations. During the introduction of He, the chamber pressure increased to $2\times 10^{-3}\;torr$. In total, 300 consecutive laser shots were applied to the target by using a Nd:YAG laser (λ=532 nm, $\tau_{p}=5-7$ ns, repetition rate = 5 Hz). The laser beam was focused into a Cu target with a beam size of 1 mm, and the Cu target was ablated by using various laser pulse energies of 30, 60, and 90 mJ/pulse, giving laser fluence of 3.8, 7.6, and 11.5 J cm^−2^ [35]. The minimum laser fluence required to initiate the ablation of Cu, termed the ablation threshold fluence ($F_{th}$), was determined to be ~3.3 J cm^−2^ in accordance with the following equation [36,37]:

$$F_{th}=\rho L_{v}a^{1/2}\tau_{p}^{1/2}.$$

where *ρ*, $L_{v}$, and a are the density, latent heat of evaporation, and thermal diffusivity of the Cu target (as listed in Table 2), and *τ_p_* is the laser pulse duration.

The structural and morphological changes in the Cu surface due to the laser irradiation were examined via scanning electron microscopy (SEM, Coxem CX-100S, Coxem Co., Ltd., Republic of Korea) and by X-ray diffraction (XRD; Philips X’PERT-PRO PANalytical, UK). The latter was performed in the 2θ range of 20 to 90° under Cu Kα radiation at the wavelength (λ) of 1.54 Å. The surface hardness of the Cu before and after the various experiments was measured by using a Micro-Vickers hardness testing machine (Autovick HM-200, Mitutoyo, Japan). In this procedure, a 0.03 kg load was applied to seven randomly selected points on each sample for approximately 30 s at room temperature. For the XRD analysis, we focused on the irradiated surface, particularly at the center of the laser spot. Regarding the hardness measurements, we took readings from seven different points, ranging from the periphery to the center of the irradiated area, and then calculated the average.

## 3. Results and Discussion

### 3.1. Surface Morphology

**Figure 2:**
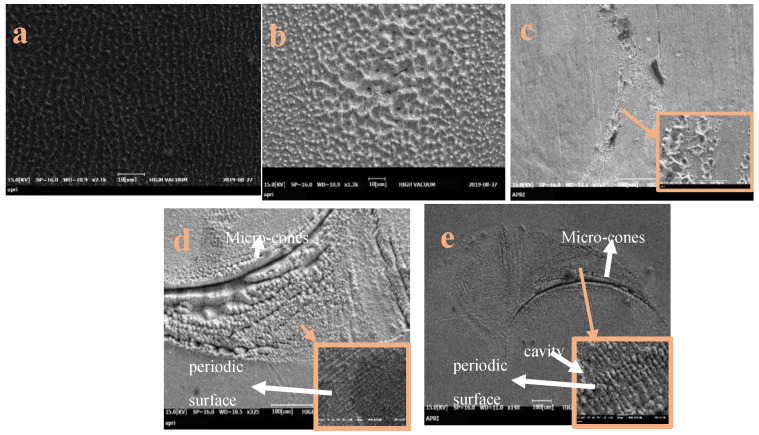
SEM images of the Cu target after irradiation at 30 mJ/pulse under (**a**) air, (**b**) vacuum, and (**c**–**e**) gas flow through the supersonic nozzle with backing pressure of (**c**) 0.5 bar, (**d**) 1 bar, and (**e**) 1.5 bar.

**Figure 3:**
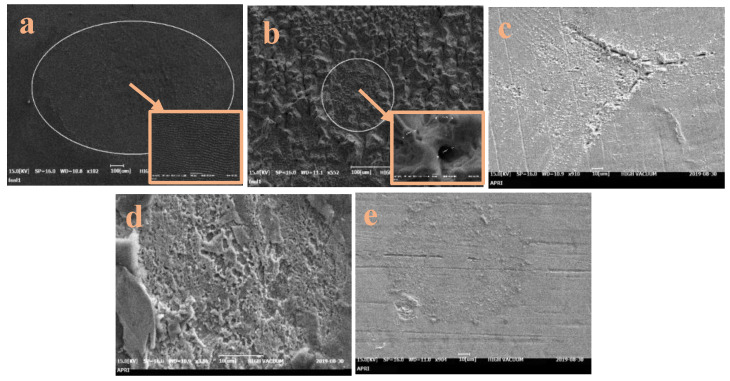
The SEM images of the Cu target after irradiation at 60 mJ/pulse under (**a**) air, (**b**) vacuum, and (**c**–**e**) gas flow through the supersonic nozzle at pressures of (**c**) 0.5 bar, (**d**) 1 bar, and (**e**) 1.5 bar.

**Figure 4:**
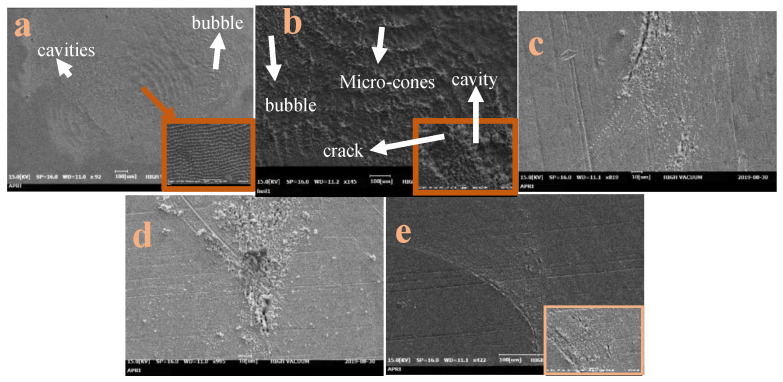
The SEM images of the Cu target after irradiation at 90 mJ/pulse under (**a**) air, (**b**) vacuum, and (**c**–**e**) gas flow through the supersonic nozzle a pressures of (**c**) 0.5 bar, (**d**) 1 bar, and (**e**) 1.5 bar.

The SEM images of the Cu surface before and after the various laser treatments are presented in Figure 2, Figure 3 and Figure 4. Thus, after irradiation under air at 30 mJ/pulse (Figure 2a), the Cu surface is uniformly covered by micro-cones, which can be compared with the smooth surface of the pristine Cu (Figure 1). When irradiated under vacuum, however, the Cu surface is covered by cavities and droplets in the submicrometer size range (Figure 2b). After irradiation under a gas pressure flow of 0.5 bar through the supersonic nozzle, the surface exhibits a combination of cone-shaped structures and splash patterns, along with small dips and droplets in the nanometer size range (Figure 2c and the inset). Upon increasing the gas pressure to 1.0 and 1.5 bar (Figure 2d,e), periodic surface structures in the form of cone-shaped ripples are formed on the 10 μm scale (insets). These ripples are accompanied by nanometer-sized droplets located around the periphery of the irradiated area. At the center of the irradiated surface, island-like structures, micro-pillars, and cavities are observed. These results demonstrate that the nanosecond laser treatment can be used to generate similar grating-like grooves to those obtained using a femtosecond laser.

After irradiation at 60 mJ/pulse under air, the Cu surface in Figure 3a exhibits a uniform morphology over the entire irradiated area. Within the laser spot area, a pseudo-periodic surface structure is observed. At the periphery, however, the surface is covered by cracks, cavities and micro-pillars. Under high vacuum, the irradiated surface entirely covered with grooves, micro-pillars and cavities with an average diameter of 2.5 μm (Figure 3b). After irradiation under a gas pressure flow of 0.5 bar through the supersonic nozzle, however, the Cu surface exhibits a similar cone-shaped pattern to that observed in Figure 2e, with perforations on the periphery (Figure 3c). In addition, micro-pillars are observed at the center of the irradiated surface. Increasing the gas pressure to 1 bar results in the removal of peels from the molten layer and the emergence of dip structures on the irradiated surface, thus leading to a rough surface with numerous pores (Figure 3d). Further increasing the gas pressure to 1.5 bar results in the removal of a significant portion of the molten layer, along with the formation of dips, as well as bubbles that cover the entire irradiated surface (Figure 3e).

When the laser energy is increased to 90 mJ/pulse, irradiation of the Cu surface under air results in a distinct, pseudo-periodic surface structure consisting of parallel lines in the central region, along with some evidence of bubble and cavity formation at the periphery (Figure 4a). Under high vacuum, however, the surface is covered by hillocks with an average size of 0.5 μm and grooves (Figure 4b). Under irradiation at gas pressures of 0.5, 1.0, and 1.5 bar (Figure 4c,d, each surface exhibits cone-shaped structures at the periphery, along with island-like structures at the center. Nevertheless, there are noticeable differences in surface morphology depending on the gas pressure. Thus, at 0.5 and 1.5 bar, the irradiated surface is covered with cavities and droplets. At 1 bar, however, most of the surface is covered by micro-pillars.

The distinctive topographical features observed on copper surfaces after laser irradiation in various environments, particularly under gas flow through a supersonic nozzle, can be attributed to several key mechanisms [38,39,40,41,42,43,44,45,46,47,48,49,50,51]. First, the high temperatures generated by laser irradiation cause the copper to boil, leading to the formation of bubbles and cavities. While some bubbles escape immediately, others become trapped, creating rounded cavities upon release [41,43]. Second, the uneven temperature distribution across the surface induces thermal stresses and shock waves, which cause cracks and grooves to form [49]. Additionally, the Gaussian intensity profile of the laser beam leads to non-uniform energy distribution, causing molten material to flow and form dips and ridges on the surface [38,40]. 

Moreover, the recoil pressure from the plasma plume generated by evaporation and ionization creates ripples and waves on the molten surface, which are preserved during rapid solidification [46,47]. Micro-cones also form as a result of rapid phase transitions between liquid and vapor in the superheated copper, followed by rapid cooling and re-solidification [50]. 

The effect of high-speed gas flow on the formation of surface micro-features is an area that needs further investigation. In our study, SEM images revealed distinct surface structures following irradiation at varying gas pressures through a supersonic nozzle. The surfaces displayed a combination of cone-shaped structures and periodic ripples, which were markedly different from those irradiated in air or vacuum. These results suggest that nanosecond laser treatment can produce grating-like grooves similar to those generated by femtosecond lasers under supersonic gas flow conditions.

Understanding these mechanisms is essential for optimizing and controlling laser-induced surface features for applications in microfabrication, surface texturing, and surface engineering. Continued research could provide valuable insights into the fundamental processes driving these effects and lead to the development of advanced laser-based techniques for fabricating tailored surface structures for various technological applications. However, more investigation is required to fully understand the role of high-speed gas flow in forming these surface micro-features.

### 3.2. XRD Analysis

The effects of the various laser treatments on the crystalline structures of the copper samples, and on the structural parameters that affect the material hardness, can be elucidated by XRD analysis. For example, Vasil’ev et al. demonstrated that laser radiation can transform the face-centered cubic structures of copper, aluminum, and silver surfaces into parallelepiped-based structures [52].

**Figure 5:**
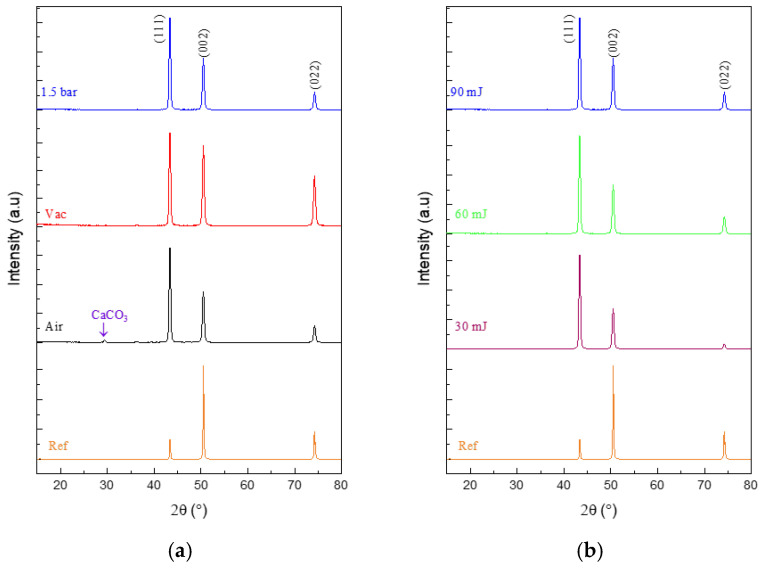
XRD patterns of the pristine Cu (Ref) and various samples (**a**) after irradiation at 90 mJ/pulse under various conditions, and (**b**) after irradiation under a high-pressure 1.5 bar gas flow at various laser energies/pulse.

The XRD patterns of the pristine and variously irradiated Cu samples are compared in Figure 5. Here, the pristine Cu exhibits peaks at 2θ = 43.405°, and 43.410°, which can be matched to the (111) crystal plane of the face-centered cubic (fcc) phase, along with peaks at 2θ = 50.554° and 50.560° due to the (002) crystal plane (ICDD card 98-065-5129). Meanwhile, the phase changes occurring in the variously irradiated samples are revealed by shifts in the peak positions and intensities. Specifically, the (111) peak increases significantly in intensity, while the (002) peak decreases, after laser irradiation at 90 mJ/pulse under air, vacuum, or at 1.5 bar (Figure 5a), and similar results are observed after irradiation under high-pressure 1.5 bar gas flow at 30, 60, or 90 mJ/pulse (Figure 5b). In addition, after irradiation under air, a new peak is observed at 2θ = 30° in Figure 5a, which can be attributed to the presence of calcite (CaCO_3_).

**Figure 6:**
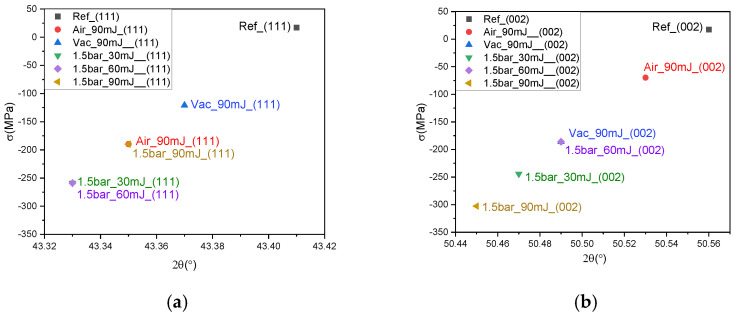
The variations in the residual stresses on (**a**) the (111) plane and (**b**) the (002) plane as a function of diffraction angle before and after irradiation under various environments.

The residual stress in the material surface plays a significant role in various fields, including structural engineering, material processing, and hardness analysis. The residual stress can be quantified and understood by using Bragg’s law to measure the variation in lattice parameters. If there is no stress present in the surface layers, the interplanar spacing between the (hkl) planes will be equivalent to that in the strain-free crystallites. Any deviation in the interplanar spacing indicates the average isotropic strain of the crystallites in the surface layers. The average stress in the surface layers can be calculated using the formula [53]:

$$\sigma =\frac{E}{2\nu }\sin\theta_{th}\left(\frac{1}{\sin\theta_{th}}-\frac{1}{\sin\theta_{exp}}\right)$$

where σ is the residual stress, E is Young’s modulus (110 Gpa for Cu), ν is Poisson’s ratio (0.35 for Cu) [54], $\theta_{th}$ is the theoretical diffraction angle, and $\theta_{exp}$ is the experimental diffraction angle. The variations in the calculated residual stresses in the (111) and (002) planes as a function of diffraction angle before and after the various laser treatments are plotted in Figure 6 and summarized in Table 3. Here, the sign of the stress value is positive for the pristine copper, but negative after the various laser treatments. This indicates a shift in the residual stress from tensile before laser treatment to compressive afterwards.

### 3.3. Hardness Analysis

**Figure 7:**
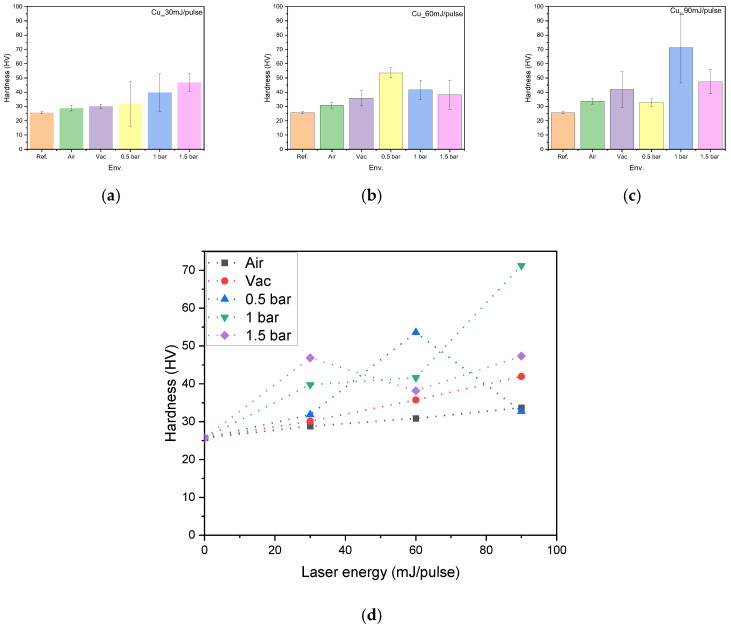
(**a**–**c**) Bar charts comparing the hardness values of the copper surface before and after irradiation under various environments at laser energies of (**a**) 30 mJ/pulse, (**b**) 60 mJ/pulse, and (**c**) 90 mJ/pulse; (**d**) plots of hardness against laser energy under each environment.

Microhardness is a critical property that characterizes the ability of a material to resist deformation under load or indentation. Among various techniques for improving the microhardness of copper, laser treatment has been found to be effective [54]. In this procedure, the laser energy and irradiation environment is known to affect the material hardness [55,56]. Previous studies have indicated that laser treatment has the capability to increase the microhardness of metals like copper by impacting various factors, including residual compressive stress, crystallite size, dislocation density, and surface structure [57,58,59]. Hence, the measured microhardness values of the pristine and variously treated samples are compared in Figure 7. When the environment is varied at an irradiation energy of 30 mJ/pulse (Figure 7a), the microhardness is seen to increase from 25.68 HV for the pristine copper to a maximum of 46.86 HV at 1.5 bar, which represents a 1.82-fold increase. This can be attributed to the development of periodic surface structures on the copper during irradiation, along with the increase in residual stress [60]. When the laser energy is increased to 60 mJ/pulse, however, the maximum microhardness value (53.56 HV) is achieved at 0.5 bar (Figure 7b), which may be due to the appearance of pores and micro-pillars on the surface. Meanwhile, at a laser energy of 90 mJ/pulse, the highest microhardness value of 71.22 HV is achieved at 1 bar (Figure 7c), representing a 2.77-fold increase compared to that of the untreated sample. This can be attributed to the presence of grooves, pits, and micro-pillars on the irradiated surface, along with the appearance of new phases (as revealed by the above XRD analysis). Furthermore, as shown in Figure 7d, the laser treatment under gas flow through a supersonic nozzle further enhances the microhardness by elevating the residual compressive stress and altering the surface structure. Here, the average microhardness value is seen to increase in proportion to the irradiation energy under the vacuum and air environments. Under irradiation at 0.5 bar, however, the microhardness initially increases gradually, and then more sharply, with the increase in laser energy up to 60 mJ/pulse, but then decreases with the further increase in laser energy. This may be because the micro-pillar’s structure is observed over the irradiated surface. Meanwhile, at 1.0 bar, the microhardness shows an initial sharp increase, followed by a very slight increase, and then a much more pronounced increase, with the increase in laser energy. This may be due to micro-pillars, droplets, micro-cones and dips structures covering the irradiated surface. Finally, at 1.5 bar, the microhardness initially increases sharply, then decreases, and then increases again, so that similar values are obtained at 30 and 60 mJ/pulse. Notably, under the conditions of 1.5 bar and 60 mJ/pulse, the surface exhibits a unique structure characterized by the formation of dips and the presence of bubbles, as revealed by the SEM image in Figure 3e. Under the conditions of 1.5 bar and 30 mJ/pulse, however, the surface exhibits periodic structures in the form of cone-shaped ripples, accompanied by the formation of island-like structures, micro-pillars, and cavities (Figure 2e). These results indicate that the hardness of the copper increases due to the generation of periodic surface structures, which is consistent with Koehler’s theoretical study [60]. Furthermore, the residual compressive stress, which can be considered to represent the bond strain, plays a role in this hardness enhancement [53].

Table 4 summarizes the hardness values of copper measured under different laser energies and irradiation environments, highlighting the significant variation in hardness based on the applied conditions.

**Figure 8:**
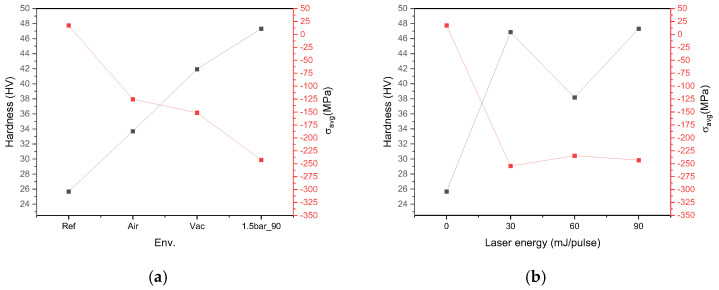
(**a**) Relationship between average residual stress (σ_ave_; red axis) and laser environment, and that between hardness value (black axis) and laser environment, at a fixed energy of 90 mJ/pulse; (**b**) the relationship between average residual stress (red axis) and laser energy, and that between hardness value (black axis) and laser energy, under a fixed environment of 1.5 bar.

The relationship between the average residual stress (σ_ave_) and irradiation environment and that between the hardness and irradiation environment are plotted on the same chart in Figure 7a. Similarly, the relationship between the average residual stress and laser energy (under a fixed environment of 1.5 bar) is plotted on the same chart as that between the hardness and laser energy in Figure 8b. Thus, it can be seen that the maximum hardness coincides with the minimum residual compressive stress, each of which is obtained at 1.5 bar and 90 mJ/pulse (Figure 8a). This is in agreement with the above XRD analysis, which indicated a strong correlation between increased microhardness and residual compressive stress, as well as revealing a shift in the residual stress from tensile before laser treatment to compressive afterwards.

Thus, the enhancement in microhardness can be attributed primarily to the presence of residual compressive stress with secondary contributions from the formation of surface structures and new phases. As noted above, the maximum microhardness value is achieved at a laser energy of 90 mJ/pulse and a pressure of 1 bar through the supersonic nozzle, which is attributed to the formation of grooves, pits, and micro-pillars on the irradiated surface, along with the emergence of new phases. Moreover, the use of a gas flow through the supersonic nozzle increases the microhardness of the material surface relative to that obtained under other environments [35]. This effect has the potential to enhance the resistance of the material towards wear and plastic deformation [53], thus making it a promising technique for various industrial applications. Hence, the present study has fully demonstrated the viability of laser irradiation under a high-pressure gas flow through a supersonic nozzle for creating surface structures on copper. These findings significantly contribute to the present understanding of laser–material interactions, and open up possibilities for the development of advanced laser-based fabrication techniques that can generate customized surface structures for diverse applications such as microfabrication, surface texturing, and surface engineering. Nevertheless, further research in this field is necessary in order to provide a deeper understanding of the underlying mechanisms and to optimize the laser-based fabrication methods. By gaining a comprehensive understanding of laser–material interactions under gas flow through a supersonic nozzle, it will become possible to devise advanced laser processing techniques that offer the precise control and tailoring of surface structures for specific applications. Furthermore, laser surface treatment shows great potential for enhancing the performance and durability of materials such as copper, enabling them to withstand harsh environments and to exhibit improved mechanical properties. Through continued research and development, laser-based techniques have the capacity to revolutionize various industries by providing innovative solutions for surface engineering and material processing.

## 4. Conclusions

Laser treatment significantly affected the surface morphology and hardness of copper, with distinct outcomes depending on the irradiation environment and laser energy. In air, the surface developed a net-like structure with micro-cones, while under vacuum, cavities and droplets were observed. When gas flow through a supersonic nozzle was used, cone-shaped structures, dips, and island-like formations appeared, with higher backing pressures enhancing surface structuring by removing molten layers and forming periodic patterns, bubbles, and pores. XRD analysis revealed shifts in diffraction peaks and the formation of new phases, highlighting microstructural changes that resulted in a shift in residual stress from tensile to compressive after irradiation. Vickers hardness testing demonstrated a 2.77-fold increase in maximum hardness under gas flow through the supersonic nozzle, attributed to residual stress, surface structures, and phase transformations. The surface and structural changes were driven by mechanisms such as boiling, thermal stress, shock waves, molten material flow, and rapid phase transitions. Overall, the results underscore the importance of optimizing laser parameters and irradiation environments to achieve the desired surface characteristics and material properties, particularly under gas flow through a supersonic nozzle.

## Figures and Tables

**Table 1:** The chemical composition (wt.%) of the copper (CU-113551) target.

C	O	Ca	Ni	Cu
9.23	2.31	3.66	1.77	83.03

**Table 2:** The thermal and physical properties of copper [36].

Material	$\rho \left(gcm^{-3}\right)$	$L_{v}\left(Jg^{-1}\right)$	$C_{p}\left(JK^{-1}{kg}^{-1}\right)$	$K\left(WK^{-1}m^{-1}\right)$	$a\left(cm^{-2}S^{-1}\right)$	$F_{th}(Jcm^{-2})$
Cu	8.96	4796	385	401	1.16	3.27

**Table 3:** The calculated residual stresses in the as-received and laser-treated copper as a function of X-ray diffraction angle.

Environment	Diffraction Plane	${2\theta }_{th}\left({}^{\circ}\right)$	${2\theta }_{exp}\left({}^{\circ}\right)$	$\Delta 2\theta \left({}^{\circ}\right)$	$\sigma \left(MPa\right)$
Ref (as-received)	(111)	43.405	43.41	+0.005	17.22
	(002)	50.554	50.56	+0.006	17.42
Air, 90 mJ/pulse	(111)	43.405	43.35	–0.055	–189.75
	(002)	50.554	50.53	–0.024	–69.73
Vacuum, 90 mJ/pulse	(111)	43.405	43.37	–0.035	–120.69
	(002)	50.554	50.49	–0.064	–186.10
1.5 bar, 30 mJ/pulse	(111)	43.405	43.33	–0.075	–258.87
	(002)	50.554	50.47	–0.084	–244.36
1.5 bar, 60 mJ/pulse	(111)	43.405	43.33	–0.075	–258.87
	(002)	50.554	50.49	–0.064	–186.10
1.5 bar, 90 mJ/pulse	(111)	43.405	43.35	–0.055	–189.75
	(002)	50.554	50.45	–0.104	–302.67

**Table 4:** Summary of copper hardness values at different laser irradiation energies and environments.

Laser Energy (mJ/Pulse)	Hardness (HV)				
	Air	Vacuum	0.5 Bar	1 Bar	1.5 Bar
Ref	25.68	25.68	25.68	25.68	25.68
30	28.78	30.10	31.82	39.77	46.86
60	30.86	35.73	53.56	41.62	38.16
90	33.68	41.94	32.72	71.22	47.31

## Data Availability

The original contributions presented in the study are included in the article, further inquiries can be directed to the corresponding author.
